# Advances in Excimer Laser Trabeculostomy within the Landscape of Minimally-Invasive Glaucoma Surgery

**DOI:** 10.3390/jcm11123492

**Published:** 2022-06-17

**Authors:** Anne Nguyen, Brian Simon, Rebecca Doan, Emily Chen, Ryan Lamrani, Jonathan Shakibkhou, Michael S. Berlin

**Affiliations:** 1 College of Science and Health, Charles R. Drew University of Medicine and Science, Los Angeles, CA 90059, USA; annenguyen@cdrewu.edu; 2 Glaucoma Institute of Beverly Hills, West Hollywood, CA 90048, USA; rebeccadoan@ucla.edu (R.D.); echen910@usc.edu (E.C.); lamraniryan05@gmail.com (R.L.); jayshakib123@g.ucla.edu (J.S.); 3 Stritch School of Medicine, Loyola University Chicago, Maywood, IL 60153, USA; bsimon1@luc.edu; 4 College of Letters and Science, University of California, Los Angeles, Los Angeles, CA 90095, USA; 5 College of Arts and Sciences, Emory University, Atlanta, GA 30322, USA; 6 Eastern Virginia Medical School, Norfolk, VA 23507, USA; 7 School of Medicine, Wayne State University, Detroit, MI 48201, USA; 8 Jules Stein Eye Institute, University of California, Los Angeles, Los Angeles, CA 90095, USA

**Keywords:** micro-invasive glaucoma surgery, minimally invasive, glaucoma, excimer laser trabeculostomy, intraocular pressure, treatment, ELT, MIGS, guidance, laser

## Abstract

Primary open-angle glaucoma (POAG) is currently treated with a variety of surgical and non-surgical approaches. Minimally invasive glaucoma surgery (MIGS) involves several devices and procedures that lower intraocular pressure (IOP) by increasing aqueous outflow. The first laser-based MIGS procedure, Excimer Laser Trabeculostomy (ELT), has emerged as a safe and effective treatment option. This article reviews ELT within the context of alternative MIGS procedures and focuses on the historical development of ELT, principles and techniques of the ELT procedure, safety and efficacy data, comparison to other outflow procedures, and future augmentations to expand the use of ELT. Performed alone or as an adjunct to cataract surgery, ELT has minimal complications and has shown long-term effectiveness in lowering intraocular pressure in thousands of patients. The non-thermal laser-tissue interactions of excimer lasers minimize peripheral tissue damage and ensure outflow channel patency without requiring foreign body implants or creating blebs. The development of 2D optical and 3D optical coherence tomography (OCT) guidance systems will eliminate the need for a goniolens to visualize angle structures and enable thousands more surgeons to perform ELT in the future.

## 1. Introduction

Glaucoma is the world’s leading cause of irreversible blindness. In the most common form of the disease, primary open-angle glaucoma (POAG), the optic nerve is damaged due to an obstruction of aqueous outflow at the juxtacanicular trabecular meshwork (TM) and the inner wall of Schlemm’s canal (SC) [1]. The obstruction causes an increase in intraocular pressure (IOP), which results in progressive vision loss. In 2020, it was estimated that there were approximately 76 million people affected by POAG. By 2040, it is projected that this number will increase to 111.8 million [2]. Some of the risk factors include family history, genetics, and age [3]. Early diagnosis and treatment are key factors in the progression and prevention of blindness for the disease [4]. The current treatments available for glaucoma include medications, lasers, and various surgical procedures. Although these options are safe and efficacious, they include many side effects and treatment regimens. In recent years, the usage of microinvasive glaucoma surgeries (MIGS) has become a more prevalent modality of treatment. These MIGS surgeries are preferable due to their minimal disruption to surrounding tissues, which allows for rapid recovery and minimize effect on patients’ quality of life [5]. One such invention, Excimer Laser Trabeculostomy (ELT), developed by Berlin et al. in 1987, is the first laser-based MIGS procedure and is advancing the modern-day treatments available for open-angle glaucoma with its safety efficacy profile and long-lasting results [6,7].

## 2. Glaucoma Treatments

Current treatments for glaucoma can be categorized into surgical and non-surgical techniques. Non-surgical treatments predominantly consist of an array of eye drops including beta-blockers, parasympathomimetic drugs, sympathomimetic drugs, alpha1-blockers, etc. that lower the IOP [8]. In addition to side effects inherent to the medications themselves, preservatives that are typically added to these eye drops are considered responsible for the multiple systemic and ocular adverse side effects reported [8]. Used in higher IOP patients in which eye drops are ineffective, acetazolamide (Diamox) is an oral medication that has also been used to treat glaucoma non-surgically [9]. Because eye drops and oral medications require regiment adherence, using them daily can be difficult for patients, for many reasons. Thus, the known noncompliance rate for ocular dosing is around 40–60% [10]. This lack of adherence puts glaucoma patients at significant risk for the ongoing loss of vision.

Surgical treatments for glaucoma thus present themselves as a more permanent and vision-preserving alternative to daily medications. Surgical treatments can be further categorized into “filtering” and “non-filtering”, which includes laser-based surgeries. Filtering surgeries, which include trabeculectomy and tube shunts, are surgical procedures that either create a flap in the sclera as well as a drainage opening within the sclera or insertion of a drainage tube in order to lower IOP [11]. Non-filtering surgeries include stent, non-stent, and laser-based procedures considered minimally invasive (or micro-invasive) glaucoma surgery (MIGS) and “office-based” laser procedures (SLT, ALT), all of which both decrease the requirement for adherence to and often the number of medications required for IOP control [12,13].

Office-based laser surgeries are typically performed when oral and topical medications and, rarely, when filtering surgeries are not effective. These include selective laser trabeculectomy (SLT) and argon laser trabeculectomy (ALT). In SLT, 532 nm laser energy is directed onto the trabecular meshwork using very short laser pulses of three nanoseconds, which minimizes thermal damage relative to ALT and enables IOP lowering to some extent. Several mechanisms for its efficacy have been proposed, however, none of them have been confirmed [14,15]. As early as 2001, Berlin proposed that SLT be considered in lieu of topical medications as first-line therapy for mild to moderate open-angle glaucoma. In 2019, Gazzard et al. (LIGHT trial) confirmed Berlin’s 2001 proposal by separating 718 newly diagnosed and untreated open-angle glaucoma patients into two treatment groups consisting of either eye drops or SLT [16]. Within those that underwent SLT compared to eye drop treatments, 78.2% of patients in the SLT group did not need drops to maintain target IOP and were within target IOP for more visits than the eye drop group. In addition to post-treatment, 11 eye drop patients required surgery to lower IOP, whereas no SLT patients needed surgery. SLT was also found to be more cost-effective than eye drops. Compared to SLT, 532 nm ALT also reduces IOP by applying laser energy to the trabecular meshwork. However, the longer pulse duration of 0.1 seconds creates thermal damage [14].

More recently, MIGS have gained popularity to treat patients with mild to moderate glaucoma due to their ability to reduce potential complications. In addition, MIGS procedures often have a higher success rate and faster recovery time. MIGS procedures include several devices and procedures that lower IOP by increasing aqueous outflow. MIGS devices include micro-stents that work to target Schlemm’s canal (iStent inject^®^) and the subconjunctival (Xen^®^ Gel Stent). MIGS procedures include canal-based disruptions (GATT), microblade (ABiC, Kahook, etc.), and laser-based ELT.

## 3. MIGS Procedures

### 3.1. Trabecular

#### 3.1.1. Trabecular Microbypass Stent (iStent, Glaukos, Laguna Hills, CA, USA)

iStent^®^ consists of an ab interno insertion of a single “L-shaped” microstent. iStent Inject^®^ consists of an ab interno insertion of two “rivet-shaped” microstents into the trabecular meshwork. This creates a bypass pathway that allows the aqueous humor to flow into Schlemm’s canal and restore the natural aqueous outflow pathway in the eye. Studies have shown that after a 3-year follow-up, patients who received a single iStent^®^ implantation subsequently incurred increasing IOP and continued reliance on additional eye drop medications after the iStent^®^ surgery [17]. Additionally, the implementation of iStent^®^ and/or iStent Inject^®^ is limited, in that they are approved only in conjunction with cataract surgery. Since the creation of the original iStent^®^, Glaukos has developed newer generations, including the iStent inject W^®^. The iStent inject W^®^’s wider diameter (360 µm) creates increased visibility and theoretically improves ease of implantation.

#### 3.1.2. Schlemm’s Canal Scaffold (Hydrus, Ivantis, Irvine, CA, USA)

The Hydrus Microstent is an 8 mm long metallic stent composed of biocompatible nitinol designed to bypass the trabecular meshwork of the eye. The Hydrus uses a preloaded delivery system that dilates roughly three clock hours of Schlemm’s canal to provide an alternate route for aqueous outflow [18]. In a study determining the efficacy of the Hydrus Microstent, 3 years after combined cataract surgery and implantation of the Hydrus Microstent, IOP was 16.7 ± 3.1 mm Hg compared to 17.0 ± 3.4 mm Hg in the group that underwent cataract surgery alone. Additionally, in the Microstent cohort, the number of glaucoma medications required after the procedure was 0.4 ± 0.8, significantly lower than the cataract surgery alone cohort (0.8 ± 1.0) [19].

### 3.2. Suprachoroidal

#### Suprachoroidal Microstent (Cypass Transcend Medical, Menlo Park, CA, USA)

Through the uveoscleral pathway, suprachoroidal MIGS procedures such as the CyPass device create direct access from the anterior chamber to the suprachoroidal space. The CyPass microstent composed of biocompatible, flexible polyimide material was designed to be inserted into the supraciliary space to enable aqueous outflow into this space [20]. Initially, the Cypass device showed promising IOP and medication reduction post-surgery. However, 5-year data indicated that corneal endothelial cell loss was a significant complication and the Cypass was recalled in 2018 [21]. There are no current suprachoroidal MIGS devices approved in the US. However, iStent Supra and MINIject are approved and in use elsewhere.

### 3.3. Supraconjuntival Space

#### XEN 45 Gel Stent Implant (Aquesys, Aliso Viejo, CA, USA/Allergan, Irvine, CA, USA)

XEN 45 Gel Stent was designed to function similar to trabeculectomy to enable aqueous humor from the anterior chamber to drain into the suprascleral space, but in a more controlled and easily performed manner than trabeculectomy. The stent was originally intended to be inserted ab-interno across the anterior chamber into the subconjunctival space, but has evolved into either an ab-interno or ab-externo surgical procedure. In either case, the insertion of this stent enables aqueous outflow leading to the formation of a filtering bleb. However, procedures which are similar to trabeculectomy to enable bleb formation pose well-known risks for scar formation postoperatively, which require ongoing monitoring and interventions. Thus, insertion of the XEN 45 Gel Stent is often accompanied by subconjunctival injections of mitomycin C and/or 5FU. Additional intraoperative complications include the device dislodging, requiring reimplantation in a different location as well as bleeding [5]. Post-operative procedures, especially sequential needling, with and without anti-metabolites are often required after XEN 45 implantation [22,23]. In a two-year follow-up with 63 patients, it was found that 62% of the patients required needling procedures to reestablish aqueous outflow [23].

### 3.4. Non-Stent Incisional Surgeries

#### 3.4.1. Ab Interno Trabeculotomy, Trabectome Device (NeoMedix, Tustin, CA, USA)

Trabectome^®^ is an ab interno trabeculotomy device that consists of an electrocautery handpiece with irrigation and aspiration. This device aims to thermally “deroof” Schlemm’s canal and remove a segment of the trabecular meshwork and inner wall of SC in order to increase aqueous outflow and thereby reduce intraocular pressure [24]. Although initial results using Trabectome as a treatment for POAG were promising, early failures often occur due to residual and sequential scarring [25].

#### 3.4.2. Kahook Dual Blade Goniotomy (New World Medical)

The Kahook Dual Blade (KDB) is a single-use ophthalmic device designed to remove the trabecular meshwork (excisional goniotomy) and create an unobstructed pathway for aqueous humor to flow out of the anterior chamber of the eye. The KDB enters the anterior chamber via a clear corneal incision and is directed towards the trabecular meshwork. The dual blade creates parallel incisions in the trabecular meshwork. Although TM tissue is removed, superior and inferior leaflets remain, which often cause scarring [26]. In the longest (36 months post-op) published study determining the success rate of KDB, the mean IOP was reduced from 19.5 ± 6.9 mm Hg on 2.4 ± 1.4 glaucoma medications preoperatively to 11.9 ± 2.7 mm Hg on 1.6 ± 1.4 medications [27]. In another study, the initial decrease in usage of IOP lowering medications after combined phacoemulsification and excisional goniotomy with the Kahook Dual Blade was significant but did not hold consistent long-term. Over a 24-month follow-up period after the procedure, a gradual increase in medication usage was reported, although still lower than the pre-op baseline number [28]. To determine the long-term efficacy of the KDB, more studies need to be completed with longer follow-up time periods post-op.

#### 3.4.3. Ab Interno Canaloplasty (ABiC)

The Ab interno Canaloplasty is a non-penetrating surgery that aims to increase the drainage through Schlemm’s canal. A corneal paracentesis enables access to the ABiC microcatheter, which crosses the anterior chamber to access Schlemm’s canal to perform a 360° cannulation of SC, exiting through the other end of the canal opening. A stent suture is tied to the catheter and moves as the microcatheter is reversed back through SC. The suture is then knotted together in place to provide inward distension, restoring the natural aqueous outflow pathway. Common postoperative complications from ABiC include hyphema, cataract formation, IOP spikes, and hypotony [29].

#### 3.4.4. Gonioscopy-Assisted Transluminal Trabeculotomy (GATT)

In GATT, a corneal paracentesis in the superonasal or inferonasal quadrant serves as the entry site for an illuminated microcatheter which is inserted across the anterior chamber into Schlemm’s Canal under goniolens visualization. Microforceps are used to grasp the microcatheter within the AC to insert it through the trabecular meshwork into and around SC to perform a 360° cannulation. The illuminated tip of the catheter is then externalized from the same initial corneal incision and, grasping both ends of the microcatheter, is pulled to create a 360° ab interno trabeculotomy, tearing the inner wall of Schlemm’s Canal and the trabecular meshwork. Opening the entire trabecular meshwork drainage system leads to an increase in aqueous outflow and lower IOP [30]. In a study conducted by the Glaucoma Associates of Texas, the most common postoperative complication was hyphema, which was seen in 30% of patients after 1-week [30].

#### 3.4.5. Endoscopic Cyclophotocoagulation (ECP)

Endoscopic cyclophotocoagulation (ECP), in contrast to outflow enabling MIGS procedures, is intended to decrease the production of aqueous humor by thermal photocoagulation rather than increase outflow. An endoscopic probe, containing both visualization and treatment fibers, is inserted through a temporal or superior clear corneal incision and positioned near the pupillary border. Separate fibers within the probe aid in image guidance, use a 175 W xenon light as an illumination source and use an 810 nm diode laser for photocoagulation. This thermal laser beam is focused on ciliary processes to ablate these structures, causing thermal shrinkage, tissue destruction evidenced by whitening, and subsequent decreased aqueous humor production, which results in decreased IOP [31]. Due to the invasive nature and difficulty in the titration of the effect, ECP is often reserved for late-stage glaucoma treatments only and limited to pseudophakic or aphakic patients [32].

## 4. Excimer Laser Trabeculostomy (ELT)

### 4.1. Development of ELT

Excimer Laser Trabeculostomy (ELT), the first clinically validated laser-based MIGS procedure, utilizes an excimer laser to create channels connecting the anterior chamber to Schlemm’s canal. ELT was developed concurrently with the application of excimer lasers for corneal refractive surgery. Excimer lasers, consisting of noble gases in their halogenated forms, were developed by Nikolay Basov in 1970. These lasers were first pioneered in ophthalmology in the 1980s for corneal surface procedures such as photorefractive keratectomy [33]. The first known application of excimer lasers in glaucoma filtering surgery was performed in 1987 when Berlin et al. created full-thickness scleral fistulas ab interno using a transanterior chamber fiber-optic delivered 308 nm xenon chloride excimer laser in rabbit eyes. This research enabled remarkable long-term IOP lowering in the rabbit eyes, demonstrating that the photochemical laser-tissue interaction with the excimer laser, which minimizes collateral thermal damage, was unlike photothermal or photodisruptive lasers [34].

The ELT procedure evolved from these full-thickness fistula procedures to create ab interno channels that end within Schlemm’s canal, e.g., trabeculostomy, due to the precision of excimer laser/tissue interactions in which tissue excision depth could be precisely calculated [35]. These laser/tissue interactions are remarkably dissimilar to those of the frequency-doubled Nd:YAG laser (532 nm) used for SLT. Unlike SLT and ALT, the excimer laser has the advantage of nonthermal submicron tissue excision precision, which minimizes trauma to the fibroblast-containing outer wall of Schlemm’s canal, thus decreasing scar-forming inflammatory responses and thereby ensuring longevity while concurrently improving outflow [36]. While the 193 nm excimer laser is ideal for corneal surface ablation, this wavelength is not fiber-optically transmissible. Thus, the 308 nm excimer laser, which is fiber-optically transmissible, enables intracameral procedures [35]. This approach to bypass outflow obstruction is similar to stent-based MIGS but does not require foreign body implants, e.g., stents. Because the laser-tissue interaction is essentially non-thermal, the channels created remain patent.

Vogel and Lauritzen were the first to perform ELT clinically in 1996, thus the first MIGS [37]. The procedure has been approved for use in the European Union and Switzerland since 1998, with clinical studies pending in Canada and the United States. In addition, new guidance technologies are being developed.

### 4.2. The Principles and Techniques of ELT

ELT (earlier known as excimer laser trabeculotomy) utilizes a 308 nm xenon chloride excimer laser to precisely create channels between the anterior chamber and Schlemm’s canal to increase outflow channels and lower IOP for open-angle glaucoma. The ab interno, non-thermal approach protects the integrity of adjacent tissues and collector channels with minimal scar formation and inflammatory responses [35,38]. Additionally, since ELT uses a laser, there are no foreign bodies or devices implanted, lowering further risks of complications. In addition, pneumatic canaloplasty, the pressurized gas produced during the tissue ablation process, may further enable the dilation of Schlemm’s canal and may augment IOP lowering. This procedure has been performed stand-alone and also concurrent with phacoemulsification. It is titratable and repeatable (although current long-term data documents repetition has been rarely necessary) [39].

Procedurally, prior to ELT surgery, the patient is given topical, peribulbar, or retrobulbar local anesthesia [35,39]. After paracentesis and stabilization of the anterior chamber with viscoelastic, the surgeon, under gonioscopic or endoscopic guidance, inserts a fiber optic probe across the anterior chamber to contact and minimally compress the trabecular meshwork [35]. Prior to probe insertion, the laser is automated to initially calibrate fluence for each single-use disposable fiber. Following insertion into the eye, the laser is surgeon-activated to deliver an adequate number of laser pulses at each treatment site to enable entry into the inner wall of Schlemm’s canal. The probe is repositioned for each channel until, under current protocol, 10 (approximately 180-micron diameter) channels are created [35,39]. The probe is then removed and viscoelastic is replaced with BSS (irrigation). Post-operatively, steroids and antibiotics are administered, and the eye is protected by a shield or patch. The patient’s postoperative IOP is subsequently monitored [35].

### 4.3. ELT Safety and Efficacy

Data from multiple studies provide evidence for the safety and efficacy of the ELT procedure. The first ELT study conducted on human eyes was in 1996. The preliminary results found that IOP was reduced by 11 mm Hg over a follow-up time of 5 months for four out of six patients [37]. In a study by Vogel and Lauritzen (1997), in 22 out of 27 eyes, it was found that IOP was reduced by 7 mm Hg. Although 12 of the eyes continued to require medications, lower doses were needed and the patients maintained lowered IOP levels [40]. A subsequent one-year study that followed 166 eyes post-op found that IOP decreased from 26.4 ± 6.2 to 16.9 ± 4.7 mm Hg [41]. A five-year study completed on 46 eyes found that after 5 years, IOP decreased from 25.5 ± 6.3 to 15.9 ± 3.0 mm Hg and the number of IOP lowering medications decreased from 1.9 ± 0.9 pre-op to 0.9 ± 1.1, further confirming longevity [42]. Complications from ELT are rare and include short-term microhyphema common to all canal-based MIGS procedures which are clinically insignificant. The IOP lowering efficacy and decrease in medical therapy validates that ELT, both as a stand-alone procedure and also when combined with phacoemulsification, has been shown to be safe and efficacious with fewer side effects than other MIGS or more invasive procedures such as trabeculectomy [41,43,44].

Phacoemulsification (phaco), a surgical treatment for cataracts, can readily be combined with ELT. Multiple independent studies validate the benefits of combining ELT with phaco. One such study by Töteberg-Harms (2011) followed 28 eyes of 28 patients who underwent ELT + phaco. After one year, the study found that IOP was reduced by 34.7% and medications were reduced on average by 0.79 [45]. Another study by Töteberg-Harms (2013) of combined ELT + phacoemulsification separated patients with ≤21 mm Hg preoperative IOP (study group) from patients with >21 mm Hg (control group). ELT was effective in both groups (64 eyes of 64 patients). There was a 23% decrease in IOP and a 38.9% decrease in anti-glaucoma medication in total for both groups. IOP and number of anti-glaucoma medication for the control group was reduced by 36.6% and 29.5%, respectively, which demonstrated greater pressure and medication usage lowering than the study group in which the IOP and number of anti-glaucoma medication were reduced by 11.5% and 42.9%, respectively [46]. The researchers concluded that a significant reduction in IOP was maintained over the one-year follow-up period regardless of preoperative IOP levels. Another independent study by Moreno Valladares (2021) with a one-year follow-up of 34 eyes of 29 patients found that the mean IOP decreased from 20.9 ± 2.6 mm Hg to 16.3 ± 1.9 mm Hg at one year [44]. The number of medications decreased from 1.7 ± 0.7 to 0.3 ± 0.8 and 81% of the 34 eyes were medication free. These studies and studies of longer durations noted below provide further validation of the effectiveness and safety profile of ELT when combined with phaco.

ELT in its current state is not only safe but also efficacious in lowering intraocular pressure over the long term (data available over a 12-year post ELT period) [7]. It is minimally invasive and stealthy as it removes tissue non-thermally. When openings are made into Schlemm’s canal, as evidenced by blood reflux, they stay patent over the long term and do not heal [7,35]. There are no foreign body implants or blebs. ELT is likely a preferred option among MIGS procedures as an adjunct to cataract surgery and also has demonstrated long-term efficacy as a standalone procedure [47].

### 4.4. ELT Comparison to Other Outflow Procedures

**Figure 1:**
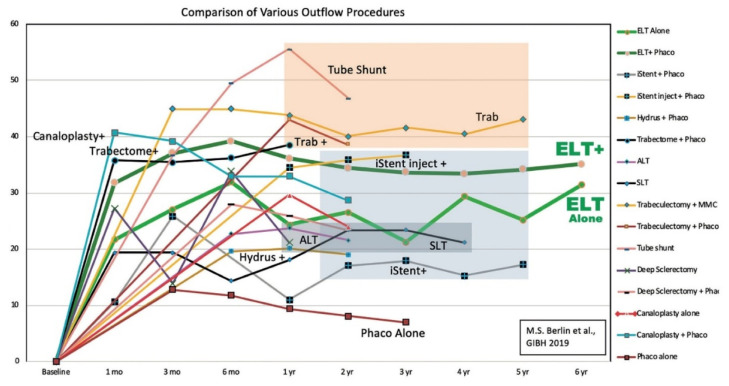
From Berlin et al. (2019): Comparison of various outflow procedures over time based on published results of exemplary studies. The *y*-axis represents the percentage decrease of IOP from Pre-Op IOP and the *x*-axis represents the number of years. Reprinted with permission from Ref. [35]. 2019, Kugler Publications.

ELT has been compared to numerous outflow procedures. Although it may not lower IOP as much as other more invasive procedures, longer-term data has shown its ability to reduce IOP to a lower and stable level over a longer time period [35]. Most notably, a 38.6% decrease after 5 years and 36.9% after 8 years [35]. When compared to more invasive procedures such as trabeculectomy and tube-shunt procedures which have more postoperative complications, ELT has minimal complications after the surgical intervention [35,42,48]. Figure 1 below summarizes the IOP for various outflow procedures over time [35].

### 4.5. Future Directions of ELT

At this time, a surgical goniolens is required to visualize the angle structures and identify Schlemm’s canal. Thus, current MIGS rely on the experience and judgment of the surgeon. It is estimated that “…MIGS is still only offered by 46% of U.S. surgeons” due to requirements for surgical gonioscopy in their (MIGS) present iterations [49].

**Figure 2:**
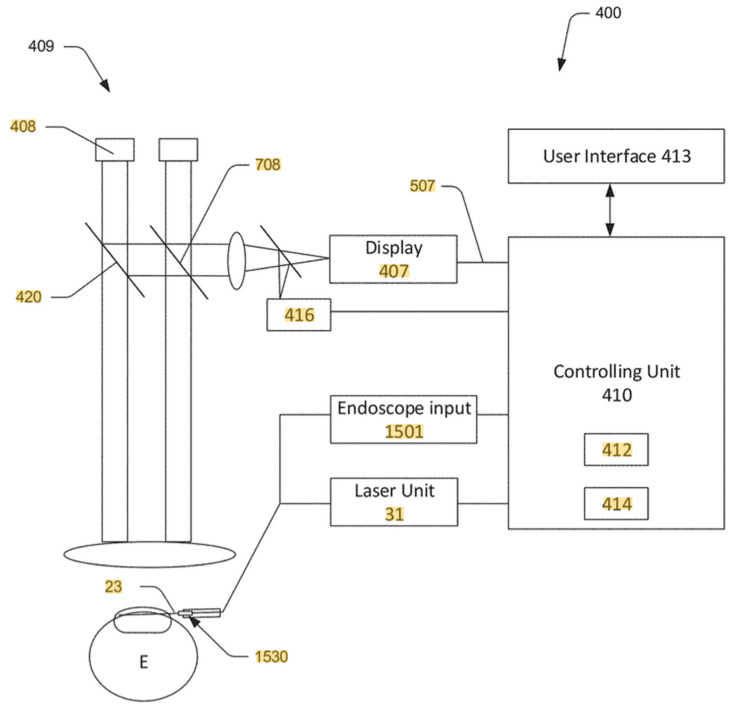
Guided Excimer Laser Trabeculostomy (ELT) GEN 1 Schematic. Berlin, MS, inventor: Image Guidance Methods and Apparatus for Glaucoma Surgery. United States patent US 11,071,647 B2. 27 July 2021.

It is anticipated that the use of ELT will grow significantly when augmented with guidance systems which eliminate the need for a goniolens by relying instead on 2D or 3D optical tools, such as an endoscope (2D) or surgical OCT (3D), to identify key anatomy during the procedure. It is estimated that a significantly greater number of cataract surgeons will be thereby enabled. See Figure 2 below.

**Figure 3:**
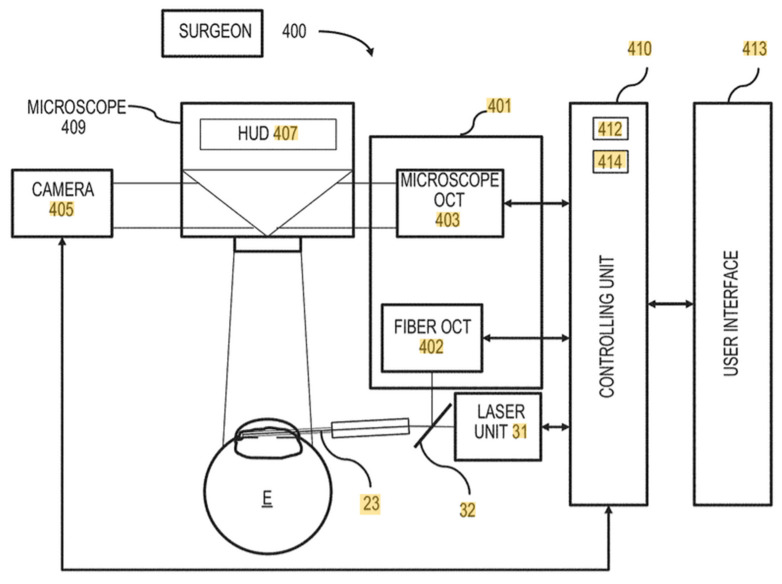
Guided ELT GEN 2 Schematic. Berlin, MS, inventor: Methods and Systems for OCT Guided Glaucoma Surgery United States patent US 10,517,760 B2. 31 December 2019.

Subsequent guided ELT, which includes enhanced 3D optical tools such as optical coherence tomography (OCT), will enable even greater precision and especially reproducible consistency for every operation [50]. Eliminating the goniolens, which is enabled by these 2D optical and 3D OCT guidance systems, is anticipated to enable a significant increase in the number of doctors comfortable utilizing this enhanced technology to better control IOP in their glaucoma patients. See Figure 3 below.

## 5. Conclusions

The emergence of MIGS in their various iterations allows for many more efficacious options for glaucoma treatments today. These multiple MIGS options allow the surgeons and their patients to determine the most safe and effective individualized treatment. ELT has a high safety, efficacy, and longevity profile that makes it a promising emerging option for the future of open-angle glaucoma care. ELT is currently being used in thousands of patients in Europe and is in the process of gaining FDA approval in the United States. Future iterations of ELT technology which include 2D or 3D guidance systems, both to eliminate the need for surgical gonioscopy and to standardize the procedure, will enable more ophthalmic surgeons to comfortably use this treatment and preserve vision in many more open-angle glaucoma patients.

## Data Availability

Not applicable.
